# Supplementary material for: Identification of Freezing-Responsive microRNAs and Their Targets in Chinese Jujube by Small RNA and Degradome Sequencing
Source: Int J Mol Sci. 2026 Sep 4;27(17):7912. doi: 10.3390/ijms27177912 (PMC13566890; doi:10.3390/ijms27177912)
Supplement: Supplementary file 1 [file ijms-27-07912-s001.zip › Supplementary Material S2/novel_miR_86.pdf]

Provisional ID : novel_miR_86
Score total : 0.4
Score for star read(s) : -1.3
Score for read counts : 0
Score for mfe : 0.1
Score for randfold : 1.6
Score for cons. seed :
Total read count : 70
Mature read count : 70
Loop read count : 0
Star read count : 0

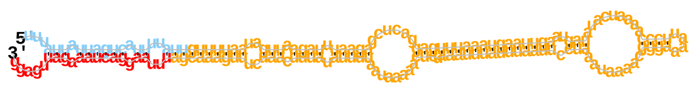

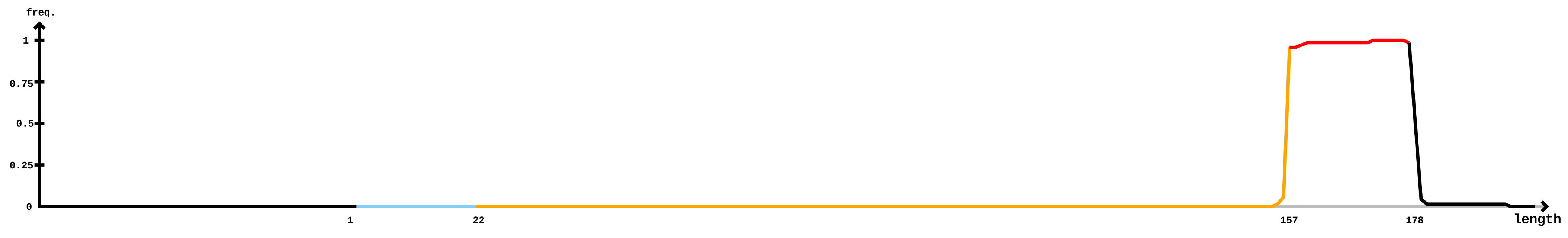

Star Mature

```
5'- auacacaguaaaucuuucauuaggacgucuuacugaauaauaaucaaaaucuuuauuauuagucauuuuauuguuuuaauaguuaagauuuaagagcucaggaguuuaaaugaauugaaugauauacuaaaacccuuaaaagggcaaaauaaauccuuaauuuauuuaaaucucaaaaauaauuuuauauuucaaucuuugaaacgauuuaaggacuaaagauugagguucugauguaaaaacuaucagg -3'  exp
    .....((((((..(.(((....))).)..))))))...........((((((.(((.((((((.((..((((((((((..(((.((((.(((((......((((((((((((((((..(((.........((((....)))).........))).)))))))))))))).))).......))))).)))).)))..))))))))))..)).)))))).))).))))))((((((....)).))))).  reads  mm  sample
    .............................................................................................................................................................................................................gauuuaaggacuaaagauugagg......................  1  0  LT-3
    ..............................................................................................................................................................................................................auuuaaggacuaaagauugagg......................  2  0  LT-2
    ..............................................................................................................................................................................................................auuuaaggacuaaagauugagguu....................  1  0  LT-1
    ...............................................................................................................................................................................................................uuuaaggacuaaagauugagg......................  28  0  LCK-1
    ...............................................................................................................................................................................................................uuuaaggacuaaagauugagg......................  29  0  LCK-2
    ...............................................................................................................................................................................................................uuuCaggacuaaagauugagg......................  1  1  LCK-2
    ...............................................................................................................................................................................................................uuuaaggacuaaaNauugagg......................  1  1  LCK-1
    ...............................................................................................................................................................................................................uuuaaggacuaaagauugagg......................  4  0  LT-2
    ...............................................................................................................................................................................................................uuuaaggacuaaagauugagguu....................  1  0  LT-1
    .................................................................................................................................................................................................................uaaggacuaaagauugag.......................  1  0  LT-2
    ..................................................................................................................................................................................................................aaggacuaaagauugagU......................  1  1  LT-3
    .............................................................................................................................................................................................................................auugagguucugauguaaaaacu......  1  0  LT-1
```
